# Focus issue on organic and hybrid photovoltaics

**DOI:** 10.1080/14686996.2018.1560065

**Published:** 2018-12-18

**Authors:** Roland Hany, Hong Lin, Fernando A. Castro

**Affiliations:** a Empa, Swiss Federal Laboratories for Materials Science and Technology, Dübendorf, Switzerland; b School of Materials Science and Engineering, Tsinghua University, Beijing, China; c NPL, National Physical Laboratory, Teddington, UK

The ongoing interest in organic and perovskite solar cells comes from the forecasted excellent potential for low-cost solar electricity generation that these technologies offer. Organic and perovskite solar cells can be fabricated from abundant materials with high-throughput compatible processes on flexible substrates and promise near future large-scale electricity production as well as novel and alternative applications in buildings, portable electronics or in tandem thin film devices.

Research activities on perovskite solar cells have been exploding over the last years and a simple title search including ‘perovskite solar cell’ in the web of science delivers well over 1500 hits for the time January–October 2018 alone! Likewise, organic solar cells are still in the spotlight and recent advances with ternary blends and non-fullerene acceptors allow now for power conversion efficiencies exceeding 10% almost as a matter of routine.

In this Focus Issue of Science and Technology of Advanced Materials, we present a collection of excellent research and review articles on recent milestones in materials development, device architectures and advanced characterization techniques that highlight the great progress that is being made in both photovoltaic technologies.

The efficiency of ternary organic solar cells depends on the developing nanostructured network of donor and acceptor phases during film formation. In a typical ternary system for photovoltaic applications, a third sensitizing component is added to an underlying high-performing binary system. However, finding the ideal three-component composition by trial and error is a cumbersome process. The research paper of Makha and Heier et al. [1] demonstrates a general approach to understanding the behaviour of organic ternary blend solar cells from simple thermodynamic principles. The authors show that phase diagrams are a fundamental tool to better understand and control the morphology of ternary blend, thereby optimizing the solar cell performance in a rational way.

Newman and Tsoi et al. [2] carried out a detailed study on the burn-in period of a high-performing solution-processed organic small molecule solar cell under illumination. Device performance was followed as a function of prior solvent vapour annealing time, controlling the crystallization of the donor phase. Results from grazing incident X-ray diffraction, UV-vis absorbance, Raman and photoluminescence experiments indicate a correlation between the increase in cell stability and the degree of crystallinity of the donor; however, the degradation originates not directly from the crystallinity changes but correlates with changes in molecular conformation.

Two articles are dealing with ‘photon management’ in organic and perovskite solar cells. Zhang and Toudert [3] review recent works reporting on optical management approaches for maximizing the light absorption and reducing transmission and reflection losses in perovskite solar cells. These include the design of optical cavities, the incorporation of plasmonic or dielectric nanostructures, the use of antireflection coatings and the structuration of the internal layers. This review points out that the performance of a solar cell is not only determined by the proper materials selection and fabrication method, but that optical optimization can substantially contribute to efficiency enhancement.

The article of Pascual-San-José and Campoy-Quiles et al. [4] considers that organic materials and perovskites are interesting for building-integrated applications, where the colour and aesthetic appeal of a solar panel plays an important role. The authors implemented a theoretical methodology to assess colour tuning in polymer-based solar cells. They compare quantitatively interference effects, binaries with different donors and acceptors, and ternary systems where the third component is either active or a simple dye. The concept can be applied to perovskite solar cells as well but the colour tuning capability of perovskites is smaller than for organic materials. The methodology allows also addressing the inverse problem, in which the materials and geometry that produce a targeted colour are obtained by a fitting routine.

Beyond high efficiency, other critical factors are required for a photovoltaic concept to translate from the laboratory to a real-world technology. For perovskite specifically, there is a desire to replace lead, due to toxicology concerns. The review article by Zhang and Lin et al. [5] outlines criteria for the possible replacement of lead by less toxic elements such as tin, germanium, bismuth, antimony, indium and transition metals, and summarizes the progress that has been actually achieved with partially substituted and lead-free perovskites. Tin-based perovskite solar cells with an efficiency of over 6% perform best, but still far from the over 20% efficiency level of lead perovskites. Despite the great challenges to advance the research on lead-free perovskites, the authors conclude to be confident that lead finally can be replaced by nontoxic elements in high-performing perovskite solar cells.

Shen and Catchpole et al. [6] review the use of perovskites in low-cost multi-junction tandem solar cells and solar devices. Perovskites can not only serve as the top subcell absorber for commercial solar cells, including silicon and copper-indium-gallium-selenide, but can work efficiently as bottom subcell owing to tunable bandgaps. The review gives an overview of recent progress on the main tandem structures, and describes the design improvements that have resulted in new record efficiencies. The review highlights the development of large-area tandem devices and points out critical remaining challenges, namely device stability and toxicity, which must be addressed for a successful commercialization of this technology. The opportunities of perovskites in other solar devices with a tandem configuration are described, including photovoltaic/thermoelectric generator and photovoltaic/photoelectrochemical systems.

Finally, Neukom and Ruhstaller et al. [7] present an overview of optoelectronic characterization techniques for solar cells. The techniques include J-V characteristics, charge extraction by linearly increasing voltage, transient photovoltage and open-circuit voltage decay, impedance spectroscopy and more. The interpretation of experimental results is not straightforward but quantitative solar cell and materials parameters can be obtained from a combination of several experimental techniques with numerical drift-diffusion simulation. The authors demonstrate that in many cases it is not sufficient to fit only results from one experiment to unambiguously determine physical parameters. This is because parameters can be correlated and different cell imperfections and defects can have a similar influence on the measurement result. Parameter correlations can be reduced by combining several experimental techniques. Using this approach, the authors simulate successfully results from nine experimental techniques with one, global, parameter set.

We thank all authors that contributed to this Focus Issue.
